# Levothyroxine Interactions with Food and Dietary Supplements–A Systematic Review

**DOI:** 10.3390/ph14030206

**Published:** 2021-03-02

**Authors:** Agnieszka Wiesner, Danuta Gajewska, Paweł Paśko

**Affiliations:** 1 Department of Food Chemistry and Nutrition, Faculty of Pharmacy, Jagiellonian University Medical College, 9 Medyczna, 30-688 Kraków, Poland; agnieszka.wiesner@doctoral.uj.edu.pl; 2 Department of Dietetics, Institute of Human Nutrition Sciences, Warsaw University of Life Sciences - SGGW (WULS), 159C Nowoursynowska, 02-787 Warsaw, Poland; danuta_gajewska@sggw.edu.pl

**Keywords:** levothyroxine, food, interaction, coffee, fiber, soy

## Abstract

Levothyroxine (l-thyroxine, l-T4) is a drug of choice for treating congenital and primary hypothyroidism. Although clinically significant interactions between l-T4 and food can alter the safety and efficacy of the treatment, they still seem to be generally underestimated by patients, physicians and pharmacists. This review aimed to investigate the effects of meals, beverages, and dietary supplements consumption on l-T4 pharmacokinetics and pharmacodynamics, to identify the most evident interactions, and to perform the recommendations for safe co-administering of l-T4 and food. A total of 121 studies were identified following a systematic literature search adhering to PRISMA guidelines. After full-text evaluation, 63 studies were included. The results proved that l-T4 ingestion in the morning and at bedtime are equally effective, and also that the co-administration of l-T4 with food depends on the drug formulation. We found limited evidence for l-T4 interactions with coffee, soy products, fiber, calcium or iron supplements, and enteral nutrition but interestingly they all resulted in decreased l-T4 absorption. The altered l-T4 efficacy when ingested with milk, juices, papaya, aluminium-containing preparations, and chromium supplements, as well as observed enhancement effect of vitamin C on l-T4 absorption, shall be further investigated in larger, well-designed studies. Novel formulations are likely to solve the problem of coffee, calcium and iron induced malabsorption of l-T4. Maintaining a proper time interval between l-T4 and food intake, especially for coffee and calcium, or iron supplements, provides another effective method of eliminating such interactions.

## 1. Introduction

Levothyroxine (l-thyroxine, l-T4) is a drug of choice for treating primary hypothyroidism, which in developing countries generally occurs due to Hashimoto thyroiditis, thyroidectomy, or iodine deficiency [1]. In 2020, l-T4 was the third most often prescribed drug in the USA [2]. Interactions between l-T4 and food may affect the safety and efficacy of the treatment but are still widely underestimated by patients and health care professionals. McMillan et al. [3] surveyed hypothyroid patients to determine the factors affecting l-T4 therapy. Out of 925 participants, 51.8% used dietary supplements known to interact with l-T4, especially calcium (47.5%) and iron (11.9%), whereas 68% reported frequent (more than twice a week) intake of food and beverages rich in fiber (bran flakes, fiber bars, fiber drinks, or broccoli florets), iodine (dried seaweed, cranberries, plain yogurt, cod), or soy. The disturbing findings were that 20% of patients took l-T4 together with breakfast, lunch, or dinner, and 21.5% administered the drug less than 30 min before the meal. Simultaneously, 124 patients (13.4%) experienced difficulties in controlling their hypothyroid symptoms. Thus, the authors suggest that l-T4 interactions with food, beverages, and dietary supplements, consumed by the participants, can, at least partly, explain these facts. The study performed by Michel et al. [4] also revealed the patients’ inadequate knowledge on l-T4 proper administration schedules. Forty five patients, treated with l-T4, completed a telephone survey. Out of 21 participants, 80% ingested calcium supplements within 4 h before, or after taking l-T4, whereas 67% within 1 h. Five patients reported administering iron or magnesium supplements within less than 1 h from taking l-T4. Only two of all the participants were advised to separate supplements and l-T4 intake.

This review is aimed to investigate the potential effects of meals, beverages, and dietary supplements consumption, as well as dosing regimen, on l-T4 pharmacokinetics and pharmacodynamics and to identify the most probable interactions. Moreover, some safety recommendations for co-administering l-T4 with food were suggested and performed in the review. Knowledge of how to avoid interactions may improve not only the efficacy and safety of l-T4 treatment, but also contributes to a better patient’s compliance.

## 2. Results and Discussion

### 2.1. Eligible Studies

**Figure 1:**
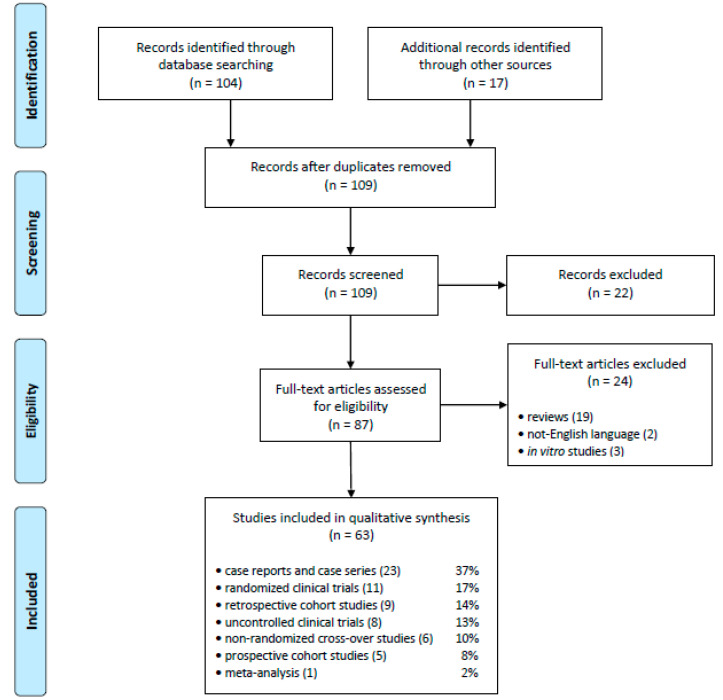
PRISMA flowchart.

As presented in Figure 1, a total of 121 articles were initially identified. After removing 12 duplicates, titles and abstracts of 109 studies were screened independently by two researchers (AW and PP), and 22 articles were excluded as not meeting the inclusion criteria, thus leaving 87 full-text articles that were assessed for eligibility by the same reviewers, resulting in a further 24 articles being removed according to the exclusion criteria (19 review papers, two not written in English and three in vitro studies). Finally, a total of 63 studies were included in the qualitative synthesis. 

### 2.2. Aspects of Levothyroxine Pharmacokinetics

l-T4 is absorbed in the small intestine, more precisely in jejunum and ileum, and to a small extent also in the stomach [5]. In patients with short bowel syndrome l-T4 absorption may be reduced due to increased intestinal passage [6]. 

Pharmacokinetic parameters of l-T4 differ in euthyroid and hypothyroid subjects. In patients with hypothyroidism, time to achieve C_max_ (t_max_) delays from 2 h to 3 h, the volume of distribution decreases from 14.7 l to 11.6 l, and the bioavailability can be higher than standard 60–80% [6,7,8]. 

Concomitant gastrointestinal diseases, such as celiac disease, *H. pylori* infection, lactose intolerance, inflammatory bowel disease, and even parasitic infestation (*G. lamblia*), etc. may cause l-T4 malabsorption [5,6].

Increased gastric pH is known to alter l-T4 absorption as well [9]. Several studies confirmed that patients with impaired gastric acid secretion, either due to the disease, or using pomp proton inhibitors (PPI), may need higher l-T4 doses to achieve the desired TSH level [10,11]. The interaction is clinically significant for chronic PPIs administration [12]. Pantoprazole and esomeprazole do not seem to alter pharmacokinetic parameters of l-T4, though the evidence is insufficient to prove its safety [13,14]. 

Apart from the tablets (Euthyrox, Levoxyl, Synthroid), l-T4 is also available in other forms such as soft gel capsules (Tirosint), oral liquid (Tirosint-SOL), and powder to prepare an intravenous solution (Synthroid). Changing the drug formulation may solve the problem of l-T4 malabsorption caused by the diseases, increased gastric pH, and drug-food interaction related factors as well. 

Liquid l-T4 is absorbed faster than its solid form [15], it was also found to be less dependable on gastric pH and conditions causing malabsorption [9,16]. Liquid l-T4 lowers TSH levels more effectively in comparison to the tablets with the same dose, both in patients with and without malabsorption [16,17,18]. Switching from the tablets to the oral liquid formulation has improved l-T4 treatment efficacy in 24 patients ingesting PPI [19] and 11 ingesting other interfering drugs [20].

l-T4 in soft gel capsules dissolves better in increased pH [21] as well. This formulation, when compared to the tablets, can improve TSH levels both in patients with gastric diseases (*H. pylori* infection, chronic gastritis, etc.) and with no proven malabsorption [22,23].

### 2.3. Schedules of Levothyroxine Administration

Previously recommended administration of l-T4 preparations included taking the drug upon waking up, due to circadian variability in TSH levels, namely higher level in the morning [24]. However, recent studies have indicated that ingesting l-T4 in the evening may be an equally effective regimen. In a randomized study conducted on 84 patients, Skelin et al. [25] compared three different timing regimens for l-T4 administration: (1) 30 min before breakfast, (2) 1 h before lunch, and (3) at bedtime, more than 2 h after dinner. TSH, free T4 (fT4), and free T3 (fT3) levels were measured at the beginning and after 8 weeks of each regimen. The results were comparable with baseline in all 3 groups. The authors concluded that different timing regimens for l-T4 are equally effective. Similar results were obtained in many other studies [26,27,28,29,30]. The decrease in bowel movements at night, and the resulting increase in intestinal absorption of l-T4 may be a possible explanation of the described observations [31]. A recent meta-analysis [32] confirmed, that l-T4 ingestion at bedtime is as effective as the one before breakfast. An evening administration may reduce the risk of drug-food interactions and enhance the patient’s compliance with the treatment. 

### 2.4. Impact of Food Intake on Pharmacokinetics and Pharmacodynamics of Levothyroxine

l-T4 absorption and bioavailability, with regard to the food, depend on the drug formulation (tablets, an oral liquid form, or soft gel capsules). In some countries tablets are the only available l-T4 formulation.

#### 2.4.1. Tablets

l-T4 is absorbed from the tablet within 20–30 min after ingestion; it takes about 3 h to complete the absorption process [7]. In the presence of food, t_max_ delays, and the peak value of l-T4 absorption decreases [6,33], similarly to drug bioavailability, from 15 to as much as 40%, depending on the study [34,35]. Lamson et al. [35] administered a single dose of 600 µg l-T4 to 48 euthyroid volunteers, either at breakfast, or 30 min before it, with 35 day washing period between both administration regimens. Breakfast consisted of eggs, bacon, toast, hash brown potatoes, milk, providing 950 kcal, 16% protein, 26% carbohydrate, and 58% fat. The authors observed significantly decreased AUC_0-48h_ (by 38–40%) and C_max_ (by 40–49%) in participants taking l-T4 with food [35]. 

The concomitant ingestion of l-T4 with food affects not only drug pharmacokinetics but also the efficacy of the treatment (measured by the changes in TSH, fT3, and fT4 levels). Seechurn et al. [36] evaluated the effect of changing l-T4 administration to 45–60 min before breakfast on elevated serum TSH levels. In all 10 patients who started to ingest l-T4 (in a medium dose of 175 µg) while fasting, TSH levels decreased significantly (by 40–96%), while the increase in fT4 levels was observed after 2 months. The results of this study, along with several other [33,37] support the recommendation to postpone food by at least 30–60 min after l-T4 tablet ingestion [5,6]. 

Perez et al. [38] conducted a randomized study on 42 hypothyroid patients, to compare l-T4 administration (in a daily dose of 98.3 ± 35.2 µg) while fasting and with breakfast. Patients consumed mostly: coffee (88.1%), white sugar (81.0%), whole milk (71.4%), white bread (69.0%), margarine (59.5%), cheese (23.8%), savoury biscuits (16.7%), non-fat milk (11.9%), whole wheat bread (9.5%), and fruits (9.5%). A standard breakfast provided approximately 162-381 kcal and consisted of 57.5% carbohydrates, 28.4% fat, 14.1% protein, and 254.1 ± 62.6 mg calcium. The fiber consumption was insignificant. TSH levels were measured at the beginning of the study and on 45, 90, 135, and 180th day. Administering l-T4 tablets with breakfast resulted in significantly higher TSH levels (2.89 ± 2.82 vs. 1.9 ± 1.76 mU/L). However, Perez et al. [38] concluded that the intake of l-T4 tablets with food can be safe and well-tolerated alternative for non-adherent patients, though it requires more frequent monitoring of TSH levels. On the contrary, patients in whom even small variations of TSH level are dangerous (i.e. pregnant women, patients with cardiac disease, or thyroid cancer) should avoid taking l-T4 with meals [38].

#### 2.4.2. Liquid Form

In vitro studies proved the stability of liquid l-T4 formulation in beverages such as milk, tea, coffee, coffee with milk, and orange juice [39]. The liquid form has also a faster onset of absorption, compared to tablets (AUC_0-2h_ (ng*h/mL): 99.1 ± 22.7 vs. 68.4 ± 32.8; t_max_ (h): 1.96 ± 1.07 vs. 2.25 ± 0.99). Greater early exposure and faster time to maximal concentration can minimize the risk of drug-food interactions [15].

Marina et al. [40] examined fT4 levels in 14 patients taking 200 µg of oral liquid l-T4; seven of them administered l-T4 while fasting, and seven with breakfast consisting of six cookies (132 kcal, 9.1% fat, 76.9% carbohydrate, 7.7% protein, 3.8% fiber) and one cup of espresso or cappuccino, both with 5 g of sucrose. The results were comparable in both groups. In another study on 59 hypothyroid patients, Morelli et al. [41] found no significant differences in the TSH level when administering liquid l-T4 with breakfast, or 10 and 30 min before (1.52 ± 0.73 mU/L, 1.46 ± 0.81 mU/L, and 1.25 ± 0.7 mU/L respectively). 

Cappelli et al. [42] conducted a randomized, double-blind, placebo-controlled trial on 77 hypothyroid patients. They assessed whether the patient’s usual breakfast (mixed with tea, coffee, milk, cappuccino, orange juice, etc.) may influence liquid l-T4 absorption. Serum TSH, fT4, and fT3 levels were comparable in patients administering liquid l-T4 formulation (in a median dose of 75 µg) with breakfast, and 30 min before. The authors concluded that liquid l-T4 can be ingested directly with the meal. Pirola et al. [43] got similar results for the same median dose of l-T4 (75 µg), on an extensive set of 761 patients. 

A possibility to administer liquid l-T4 with food may have a positive influence on patient compliance and well-being [44]. Among 102 patients, dissatisfied with their therapy with l-T4 tablets (in a mean dose of 88 ± 34.7 µg), taken before the meal, 66.6% reported improvement in the quality of life and better adherence after switching to liquid l-T4 ingested with breakfast [45]. Treatment with liquid l l-T4 formulation can be considered also in patients who are willing to keep their daily habits [46].

#### 2.4.3. Soft Gel Capsule

We found only one study [47] investigating how meals affect absorption of l-T4 in a soft gel capsule formulation. In a group of 60 euthyroid patients taking oral liquid l-T4 with breakfast, the drug was switched to the soft gel capsule form, without a change in a mean dose (106.25 ± 24.28 µg/day). Each patient maintained his dietary habits; their breakfast commonly included biscuits, yogurt, fibers, milk, coffee, tea, and orange juice. Cappelli et al. [47] measured TSH, fT4, and fT3 levels in all the patients at the beginning and after 6 months of treatment with a new formulation. No significant difference in the TSH level was found; fT4 and fT3 levels were slightly but considerably lower in patients treated with l-T4 in soft gel capsule form (by an average 7% both). The authors concluded that soft gel formulation can be taken during breakfast. Nevertheless, it is oral liquid form rather than soft gel capsules that is preferred for maintaining a stable fT4 and fT3 levels in patients with thyroid cancer or cardiomyopathy [47].

### 2.5. Levothyroxine–Fiber Interaction 

To deal with hypothyroidism symptoms, like overweight, obesity, or constipation patients often introduce a fiber-enriched diet, or administer dietary fiber supplements, without consulting their doctor. The diet type may, however, significantly influence the bioavailability of l-T4.

l-T4 non-specifically adsorbs to the fiber, what leads to the malabsorption of the drug [48]. Additionally, products that contain insoluble dietary fiber intensify bowel movements, and in consequence, intestinal absorption of l-T4 could be altered [33]. 

Liel et al. [49] described cases of 13 hypothyroid patients in whom the ingestion of fiber-enriched products (e.g. whole wheat bread, bran, granola, psyllium) led to a significant decrease in the efficacy of l-T4 tablets (in dosage range 50–470 µg/day). The authors suggested monitoring TSH levels in patients following diet modifications and increasing the dose of l-T4 when necessary. 

Chiu et al. [50] assessed the effect of pharmacological fiber supplements on l-T4 absorption in 8 volunteers, who ingested 600 µg of l-T4 tablets alone or together with 3.4 g psyllium. The l-T4 absorption was expressed as a percentage of the administered dose. The observed decrease in drug absorption, due to the simultaneous ingestion of psyllium, reached only 9% (89% vs. 80%). The authors considered this result as clinically insignificant and concluded the psyllium fiber not to be likely to alter l-T4 tablets bioavailability.

### 2.6. Levothyroxine-Soy Products Interaction 

In the recent systematic review by Otun et al. [51] it was concluded that soy supplementation had no effect on thyroid hormones (T3 and T4), though it slightly increased TSH level (with unknown clinical significance). Here, the presented data show possible interaction of soy active components: soy protein and soy isoflavones, with l-T4 treatment. Since the mid-1960s, several reports suggested that feeding soy-based formulas to infants with congenital hypothyroidism may lead to malabsorption of l-T4 tablets, and increased TSH levels [52,53,54,55]. Conrad et al. [56] confirmed the negative influence of soy isoflavones on the l-T4 treatment efficacy (measured by TSH level) for a group of 78 infants with l-T4 congenital hypothyroidism. The authors emphasized the necessity for controlling the levels of thyroid hormones to adjust l-T4 doses in patients receiving soy formulas. 

Bell et al. [57] reported a case of a 45-year-old woman after thyroidectomy—treated with 200 µg of l-T4 in tablets—who regularly ingested a soy protein-containing cocktail together with l-T4. In consequence, her fT4 and TSH levels became elevated; she also required higher l-T4 doses to achieve euthyroidism. Free T4 and TSH levels became normalized after advising the patient to separate the soy protein and l-T4 intake. The observed interaction occurred due to the adsorption of l-T4 on the surface of soy protein.

Soy isoflavones-containing supplements are widely used by women to relieve menopausal symptoms. Persiani et al. [58] conducted a randomized controlled trial on 12 post-menopausal women with hypothyroidism. They investigated the effect of soy isoflavones on bioavailability of l-T4 tablets. Patients received a supplement containing 60 mg of soy isoflavones (>19% of genistein and daidzein) with, or 6 h after, l-T4 in a daily dosage that ranged between 25–125 µg. The authors found no significant changes in l-T4 pharmacokinetics when administered with soy isoflavones.

### 2.7. Levothyroxine–Milk Interaction 

We found only one study where the influence of milk on l-T4 absorption was investigated. Chon et al. [59] administered 1000 µg of l-T4 tablets alone or together with 355 mL of 2% cow milk (containing 450 mg of calcium) to 10 healthy patients. Then they measured peak serum TT4 concentrations and AUC, both parameters were significantly lower in patients ingesting l-T4 with cow milk—by 7.8% and 8%, respectively. The results of this research cannot be extrapolated to plant-based milk alternatives (such as almond, oat, coconut, or rice milk), due to different concentrations of protein and calcium. The separation time needed to avoid possible l-T4 and milk interaction is still unknown. 

### 2.8. Levothyroxine–Coffee Interaction

Several studies on patients with hypothyroidism revealed that coffee could decrease the efficacy and safety of l-T4 treatment. The proposed mechanism for this interaction was the sequestration of l-T4 by coffee and in consequence, altered intestinal absorption of the drug [60]. Benvenga et al. [61] investigated the influence of espresso (without milk or sugar) when co-administered with l-T4. Six hypothyroid and nine healthy women were administered two 100 µg l-T4 tablets swallowed with (1) coffee, (2) water, or (3) water followed by coffee 60 min later. Th authors measured average and peak incremental rise of serum T4 concentrations and time to reach maximal serum level. Compared to water, coffee significantly lowered the incremental rise of serum T4 level, both average (by 36% in thyroid patients and 29% in volunteers) and peak (by 30% and 19%, respectively). It also significantly delayed time to reach maximal serum level (by 38 and 43 min). As no significant difference was found between groups (2) and (3), it was suggested that 1h break between coffee and l-T4 is enough to prevent the interaction. Additionally, an in vitro study found coffee to be 2-times weaker than other agents, such as antacids and fiber, known to interfere with l-T4 absorption. Sindoni et al. [62] presented cases of 6 patients in whom serum TSH level failed to be normalized or suppressed. All patients, within 1.6–2.2 µg/kg daily dosage range, declared to take l-T4 tablets together with, or shortly before, their morning espresso or barley coffee. Advising patients to separate coffee and l-T4 by 1 h, helped them to achieve euthyroidism. 

Recently, the interaction of l-T4 with American coffee (drip coffee) was also reported. Węgrzyn [63] described a case of a 52-year-old woman who developed clinical signs of hypothyroidism after taking in the morning 175 µg of l-T4 in tablets, with a cup of drip coffee. The patient was advised to postpone drinking coffee by 1h after taking l-T4. Her TSH levels normalized in 6 weeks (from 8.27 to 0.24 mU/L).

The above cases refer to l-T4 in tablets. Recent studies suggest that coffee-induced malabsorption of l-T4 can be reduced by replacing the tablets with soft gel capsules or liquid form. 

Vita et al. [64] assessed a 6-month study on eight patients with coffee-associated l-T4 malabsorption. Participants were switched from the tablet form to the soft gel capsule without a change in l-T4 daily dose; the dosage ranged from 1.6 to 2.8 µg/kg. For the first 3 months, patients swallowed the capsule with water, and had their coffee 1 h later. On days 91–180 coffee was administered less than 5 min after l-T4. TSH levels, measured at the end of each part of the study, were comparable, regardless of the time between administering coffee and l -T4 soft gel capsules. 

Cappelli et al. [65] obtained similar results for the liquid form of l-T4. The authors identified 54 patients taking oral liquid l-T4 (mean dosage: 73.15 ± 17.41 µg/day) with breakfast and morning coffee. Following evaluation of TSH, fT4, and fT3, they advised patients to consume l-T4 at least 30 min before breakfast. The tests were repeated 3 and 6 month later, and no significant differences in thyroid hormones concentrations were found. 

### 2.9. Levothyroxine-Fruit Interaction

#### 2.9.1. Fruit Juices

Different categories of transporters contribute to carrying l-T4 from the small intestine to the bloodstream, i.e. the organic anion-transporting polypeptide (OATP) family (such as OATP1A2, OATP1B1, OATP1C1, etc.), the monocarboxylate transporter (MCT) family or NTCP (sodium-taurocholate co-transporting polypeptide) transporters [5,66]. Active ingredients of juices—especially grapefruit, orange, and apple juice—may block the transporters [67,68]. 

Lilja et. al. [69] reported a case of a 36-year-old hypothyroid woman, successfully treated with l-T4 (100 µg daily). Her TSH level became elevated (63.7 mU/L) and fT4 concentration decreased (6.4 pmol/L) after marked consumption of grapefruit juice. Increasing the l-T4 dose to 150 µg was ineffective. After advising the patient to drink less grapefruit juice, TSH and fT4 levels returned to the normal range (TSH—0.291 mU/L, fT4—17 pmol/L). 

Based on this case, in a randomized study, Lilja et al. [69] assessed the influence of grapefruit juice on the pharmacokinetics of l-T4. 10 healthy volunteers were administered 200 mL of grapefruit juice three times a day for 2 days. On day 3, they ingested grapefruit juice 1 h before, together with, and 1 h after a single 600 µg dose of l-T4. Grapefruit juice reduced L-T4 AUC only by 9% and slightly decreased C_max_ (by 11%). TSH levels measured up to 24 h were comparable to the control group. The authors concluded that the relevance of the grapefruit juice- l-T4 interaction seems to be small. Yet, the lack of significant interaction observed in that study may be due to conducting it on healthy subjects. Meyer et al. [70] found that thyroid hormones upregulate OATP2B1 expression, thus, hypothyroidism may influence juice- l -T4 interaction.

Recently Tesic et al. [71] presented a case of a 31-year-old woman, unsuccessfully treated with high doses of l-T4 alone (200–700 µg a day) or in combination with liothyronine (Novothyral 100, 3 times a day). The patient reported taking l-T4 tablets on an empty stomach but often with juice or mint tea. Few days after advising the patient to administer l-T4 with water, TSH level normalized (from initial > 100 to 9.4 mU/L), and fT3 level increased (from 5.9 to 10.8 pmol/L), as well as fT4 level (from undetectable to 7.4 pmol/L). The authors suggested that juice or mint tea interfered with l-T4 absorption in that case.

Given limited and contrary data, l-T4 -treated patients should not be discouraged from rational fruit juice consumption.

#### 2.9.2. Papaya

A sudden change in a diet may impair the effectiveness of l-T4 therapy. Deiana et al. [72] reported a case of a 37-year-old patient after thyroidectomy, euthyroid on l-T4 dose of 1.6 µg/kg, with TSH levels from 1.2 to 1.9 mU/L. The patient had his check of thyroid function 7 days after a 2- week trip to the Caribbean country. The doctor documented an unexpected increase in the TSH level (25 mU/L). The patient reported the intake of large amounts of papaya (5–6 fruit per day) during his vacations. After 45 days without ingesting papaya, serum TSH concentration returned to the reference range. To confirm the interaction between l-T4 and papaya, the patient was asked to eat the same amount of papaya, as he did during vacations. The researchers measured serum levels of TSH, fT4, and fT3 before and after 7 and 14 days of excessive fruit ingestion. They found no effect in the first week but after 14 days observed an increase in TSH level (from 0.8 to 15 mU/L) and reduction of fT4 and fT3. The patient returned to the euthyroid state after discontinuing papaya intake. 

The authors discussed possible mechanisms for interaction between l-T4 and papaya. The active principle of the fruit - papain, reduces the secretion of gastric acid (up to 48 h) and increased gastric pH is associated with lower l-T4 absorption. Other fruit ingredients (i.e., terpenoids, saponins, alkaloids, and flavonoids) have cytoprotective and antiulcer properties, and can also reduce l-T4 absorption. Furthermore, papaya contains fiber, which can bind l-T4 in the intestine at such extensive intake of the fruits [72]. 

### 2.10. Interaction of Levothyroxine with Essential and Trace Elements

Di- and trivalent elements, especially calcium and iron, may decrease l-T4 bioavailability, making treatment less effective [73,74]. The proposed mechanisms that lead to increased elimination of l-T4 involve unspecific adsorption and creating insoluble complexes in the intestine [11,75]. Thus, health care professionals should discuss with their patients the adverse effects of self-supplementation likely to occur.

#### 2.10.1. Calcium

The effect of l-T4 treatment on bone mineral density is still an open question [76] but patients with hypothyroidism, especially postmenopausal women, may often use calcium supplements to prevent l-T4 -induced osteoporosis [77,78]. In a cohort study on 20 patients with hypothyroidism, Singh et al. [77] indicated that calcium carbonate may reduce l-T4 absorption. After 3-month co-administering 1200 mg/day of elemental calcium with l-T4 in a dose of 1.0 µg/kg or greater, the patients’ fT4 levels were significantly reduced, while 20% of patients had their TSH level higher than standard. Thyroid hormones concentrations normalized after calcium carbonate discontinuation. A year later Singh et al. [79] confirmed those results for 2000 mg daily dose of calcium, acutely administered with 1000 µg of l-T4 in tablets. Interactions between calcium carbonate and l-T4 were also reported in several case studies [80,81,82,83]. 

Irving et al. [11] performed a large observational study on patients, who were prescribed l-T4 tablets at least three times in 6 months. Out of all participants, 450 co-administered calcium preparations, and 429 iron supplements. The authors analyzed TSH measurements for 1 year from starting the supplementation. Taking calcium with l-T4 resulted in a significant increase in serum TSH (up to over 5 mU/L) in 4.4% of patients. Similar results were obtained for the iron group where 7.5% of the participants had increased TSH levels. Researchers concluded that calcium and iron supplements can decrease l-T4 absorption. 

Other calcium preparations were also examined for the potential interaction with l-T4. Diskin et al. [84] compared TSH levels in 65 patients taking l-T4, in a mean dose between 95–98 µg/day, with different phosphate binders. Researchers found that administering calcium carbonate, but not calcium acetate, resulted in a significantly higher TSH level (23.8 ± 19.5 mU/L). Calcium acetate interference with l-T4 absorption was denied. Zamfirescu et al. [85] made different conclusions after comparing the effect of calcium formulations—acetate, citrate, and carbonate, each in a dose containing 500 mg of elemental calcium—on the absorption of l-T4 tablets in a dose of 1000 µg. Co-administration of each of the three calcium preparations in eight healthy adults resulted in a significant reduction (20 to 25%) of l-T4 absorption, compared to l-T4 alone. Researchers emphasized that patients should take l-T4 and all the examined calcium formulations well-separated in time. 

Morini et al. [86] performed a cohort study on a group of 50 postmenopausal, hypothyroid women. They confirmed the malabsorption of l-T4 tablets (in a daily median dose of 1.47 µg/kg) when co-administered with calcium supplements containing 600–1000 mg elemental calcium/day. The researchers observed an increase not only in the TSH level but also in blood pressure, total cholesterolemia, and fasting glycemia. The effects occurred solely for calcium supplements administered within 2 h after l-T4. 

Interaction with calcium can be avoided by switching from l-T4 in tablets to oral liquid form. Benvenga et al. [87] studied 12 hypothyroid patients on daily 1000 mg of calcium. Calcium carbonate was administered 2–4 h after two different l-T4 formulations in a median daily dose of 1.7 µg/kg. The first test, after 6–8 weeks, revealed significantly lower TSH levels with the liquid formulation compared to tablets, namely 2.15 ± 1.4 mU/L vs. 8.74 ± 7.2 mU/L. The effect lasted after a 25-week follow-up. Researchers concluded that oral liquid l-T4 is resistant to sequestration by calcium. 

Interaction between calcium compounds and l-T4 is well documented. Still, patients’ knowledge on the subject remains insufficient. Mazokopakis et al. [88] surveyed 153 patients and revealed that only 8.4% of them took the calcium carbonate separated at least 4 h from l-T4.

#### 2.10.2. Iron

Phenolic, carboxylate, and amine functional groups on l-T4 facilitate the formation of insoluble or sparingly soluble complexes with ferrous salts, and this process may alter drug absorption. 

Campbell et al. [89] observed reduced efficacy of l-T4 (in dosage range 75–150 µg) when administered with 300 mg of ferrous sulfate. After 12 weeks of study, the mean level of serum TSH increased from 1.6 ± 0.4 to 5.4 ± 2.8 mU/L in 11 out of 14 patients (79%). Clinical symptoms of hypothyroidism occurred in nine participants. l-T4 malabsorption due to co-administering ferrous sulfate was also described in several case reports [90], [91]. Shakir et al. [90] suggested that such interaction can occur despite maintaining the 4–6 h interval between l-T4 and ferrous preparation. 

Leger et al. [92] reported the case of a 60-year-old woman, successfully treated with l-T4, who became hypothyroid after starting a daily intake of ferrous fumarate. She experienced elevated TSH level (243 mU/L, reference: 0.32–5.00 mU/L), and decreased T4 serum levels (<0.52 pmol/L, reference: 11.0–23.0 pmol/L). Her thyroid function normalized after 2 months of discontinuing iron supplementation.

In a recent cohort study, Atruktsang et al. [93] investigated the time to achieve euthyroidism in 605 patients treated with l-T4 in the initial dose of 1.6 µg/kg. 97 participants (16%) needed three or more dose adjustments and were classified as a prolonged dose adjustment (PDA+ group). Compared with PDA-, PDA+ group used ferrous supplements more often (1.8% in PDA- vs. 6.2% in PDA+). The researchers concluded that iron supplementation could be associated with prolonged l-T4 dose adjustment. 

Similar to calcium, the use of an oral liquid form may correct L-T4 malabsorption caused by ferrous preparations. On a group of 8 hypothyroid subjects, Benvenga et al. [87] analysed the possibility of l-T4 interaction with iron. Patients were administered 329.7 mg of ferrous sulfate, 2–4 h after the ingestion of different l-T4 formulations in a median daily dose of 1.7 µg/kg. The authors observed a significant decrease in TSH level in patients taking oral liquid l-T4, compared to tablets (1.68 ± 0.9 mU/L vs. 8.74 ± 7.2 mU/L). 

#### 2.10.3. Aluminium

Aluminium-containing preparations are available without prescription as antacids. Several case studies reported l-T4 malabsorption due to concomitant use of aluminium hydroxide [94,95]. Liel et al. [75] studied five hypothyroid subjects balanced on l-T4. Patients were administered two aluminium hydroxide-containing gel tablets 4 times a day for 2–4 weeks. A significant increase in serum TSH, specifically from 2.62 to 7.19 mU/L, was observed during periods of aluminium hydroxide ingestion. 

#### 2.10.4. Chromium

Although the effectiveness and safety of chromium picolinate supplementation in overweight and obese people are controversial [96,97], patients with thyroid diseases may use chromium supplements to control body weight. 

We found only one study on seven healthy volunteers, testing the effect of concomitant use of l-T4 (1000 µg) and chromium picolinate (1 mg). It was revealed that chromium supplementation decreases l-T4 bioavailability by 17% [98]. 

Detailed recommendations on chromium and l-T4 intake cannot be made due to the lack of further research. Nevertheless, following l-T4 interactions with other di- and trivalent metals, patients shall be advised to delay chromium preparations use to 2–4 h after l-T4 ingestion. 

### 2.11. Levothyroxine—Vitamin C Interaction 

Increased pH in the stomach affects the absorption of l-T4, thus, it was investigated whether vitamin C, an agent known to lower gastric pH, could enhance l-T4 absorption. The data, although limited, look promising.

Jubiz et al. [99] conducted the study on 31 patients with hypothyroidism and gastritis. Participants swallowed l-T4 tablets, in a median daily dose of 100 µg, with 120 mL of water containing, or not, 500 mg vitamin C. Researchers measured serum levels of fT4 and TSH at the end of 2, 4, and 6 months. TSH level decreased significantly in all patients (with the average of 69.2%) and normalized in 54.8% of them (11.1 mU/L in a control vs. 4.2 mU/L in a vitamin C group). Free T3 and fT4 levels increased significantly in almost all patients. 

Antunez et al. [100] achieved similar results in the study conducted on 28 patients with elevated TSH levels, despite being on the l-T4 dose higher than 1.70 µg/kg. Researchers asked patients to take l-T4 tablets with 1 g of vitamin C (in effervescent tablets, dissolved in 200 mL of tap water) for 6–8 weeks. A significant decrease in serum TSH level (from 9.01 ± 5.51 mU/L to 2.27 ± 1.61 mU/L) was observed afterwards.

### 2.12. Levothyroxine—Enteral Nutrition Interaction

Reis et al. [101] conducted a multicenter study in Intensive Care Units of seven teaching hospitals in Brazil to assess the prevalence of drug-enteral nutrition (EN) interactions. They considered l-T4—EN interaction as clinically significant and one of the most frequent. 

In a former study, Dickerson et al. [102] examined 13 patients with hypothyroidism. Participants received EN with l-T4 at the same dose as before the hospitalization for 20 ± 5 days. Thyroid function tests (TFTs) were performed before co-administration and then approximately once a week. Eight patients developed hypothyroidism: subclinical (TSH —6–10 mU/L, normal fT4) or overt (TSH >10 mU/L, low fT4). Researchers recommended to perform TFTs once a week in patients receiving continuous EN and l-T4. 

Manessi et al. [103] discovered that l-T4, when combined with EN, may adsorb to enteral feeding tubes. It was proposed as the reason for the observed decrease in drug efficacy. However, Wohlt et al. [104] stated that this mechanism is probably clinically insignificant, and suggested that altered l-T4 absorption is rather due to the ingestion together with food. 

Pirola et al. [105] compared the effect of administering different formulations of l-T4, in a daily dose of 1.6 µg/kg, via an enteral feeding tube. They conducted a study on 20 euthyroid patients, one day after surgery. Nurses stopped EN for 30 min before and after administering crushed l-T4 tablets; the liquid form was placed into a feeding tube with continuous enteral nutrition. Researchers measured thyroid hormones profile before and after l-T4 treatment with both formulations. The results were comparable, so the authors concluded that EN does not affect liquid l-T4 absorption. In the survey, nurses stated also that liquid formulation is easier to prepare and administer.

In Table 1, we summarized data from the most relevant works, including number of patients, their health state, l-T4 dose and formulation, type of investigated food, and effects observed in each study.

### 2.13. Limitations of Studies

We recognized several limitations of the presented studies, listed below:

- low level of evidence: most studies are single case reports, case series, cohort studies (mainly retrospective), clinical studies without control groups (see Figure 1 and Table 2);
- small number of patients in experimental studies: ≤10 participants [36,50,59,61,62,64,75,79,80,85,87,98];
- presence of older studies: from 1960s [52], 1970s [34], and 1990s [33,49,50,53,54,75,89,90,92];
- unavailable data: in several studies the l-T4 formulation and dose were not mentioned (see Table 1) as well as dietary supplement dose [11], and quantitative and/or qualitative meal composition [33,36,41,42,47];

For l-T4 the knowledge gaps on drug-food interactions are clearly visible. Heuberger [106] and Paśko et al. [107] provided a few reasons for that, such as undefined framework study, hardly available appropriate samples, or measurement difficulties. Moreover, general lack of understanding of the clinical significance of drug-food interactions, their cost, and overall impact on the population, contribute to underestimating this problem.

### 2.14. Recommendations

Although credible data are limited, Table 2 below presents the summary of significant interactions of l-T4 and food ingredients, as well as recommendations for health professionals. 

## 3. Materials and Methods

### 3.1. Search Strategy

A systematic search of the literature was performed by two independent researchers AW and PP, in adherence to the preferred reporting items for systematic reviews and meta-analyses (PRISMA) statement. The databases searched under the paper were Medline (via PubMed), Cochrane Library, and Embase covering reports from 1965 to 2020. We applied the following keywords and phrases to complete the searches: drug name (“levothyroxine”, “l-thyroxine”,” l-T4”) in combinations with “pharmacokinetics”, “food”, “food-drug interaction”, “meal”, “fruit”, “juice”, “coffee”, “soybeans”, “milk”, “enteral nutrition”, “iron”, “aluminum”, “calcium”, “chromium”, “fiber”, “papaya”, “vitamin C”. Other resources such as drugs.com, AHFS, Google Scholar, Medscape, Trip Database and UpToDate, were also researched, as well as product characteristics for the registered l-T4 formulations. Additional publications were found by checking the reference lists.

### 3.2. Inclusion and Exclusion Criteria

All articles reporting or investigating the effect of meals, beverages, and dietary supplements, but also the dosing regimen on l-T4 pharmacokinetics and pharmacodynamics were considered for inclusion in this review, without restriction for study design, sample size or participants characteristics. We decided to evaluate all existing data to obtain sufficient results to present reliable and useful recommendation for physician, pharmacist, and patients. Exclusion criteria were: review studies, in vitro studies and articles written in languages other than English.

### 3.3. Data Extraction

The extracted data included the following information: study design, number of participants and their health state, duration of treatment, l-T4 dose and formulation, qualitative and quantitative composition of food, period between l-T4 and food consumption, and reported outcomes.

## 4. Conclusions

The results of the reliable studies (RCT and meta-analysis) proved that l-T4 ingestion in the morning and at bedtime are equally effective. There is strong evidence (from RCT) to support the conclusion, that recommended administration of l-T4 with the given food depends on its formulation. Tablets should be taken 60 min before a meal; oral liquid and soft gel capsule forms can be ingested with food if that can improve patient adherence with the treatment. 

We found limited evidence from non-randomized cross-over studies, uncontrolled clinical trials, and cohort studies, for the interactions between l-T4 and coffee, soy products, fiber, calcium or iron supplements, enteral nutrition - all resulting in decreased l-T4 absorption. 

We presented reports indicating altered l-T4 efficacy when ingested with milk, juices, papaya, aluminium containing preparations, and chromium supplements, however, the clinical relevance of these interactions should be further investigated in larger, well-designed studies. The possible enhancing effect of vitamin C on l-T4 absorption needs to be proven on a larger group of patients as well. 

Switching from tablets to novel formulations can solve the problem of coffee-, calcium-, and iron-induced malabsorption of l-T4. Maintaining an interval between l-T4 and food ingestion may also lower the risk of interactions, especially with coffee, and calcium or iron supplements.

This review may contribute to a better understanding of l-T4 -food interactions among physicians and pharmacists. We hope that this will also result in the increase in patients’ awareness of the proper use of l-T4. However, more in-depth reliable studies are necessary to shed more light to the complicated but important problem of l-T4 interactions with food and dietary supplements.

## Figures and Tables

**Table 1:** Summary of data from the most relevant studies investigating the influence of food on levothyroxine pharmacokinetics and pharmacodynamics.

Study	Participants	L-T4 Dose (µg/Day)	L-T4 Formulation	Type of Food	Observed Effect
Wenzel et al. [34]	not specified	100	Tablets	not specified	↓ L-T4 absorption (by 15%)
Lamson et al. [35]	48, healthy	600	Tablets	breakfast, 950 kcal	↓ AUC0-48 h (by 38–40%), ↓ Cmax (by 40–49%)
Perez et al. [38]	42, hypothyroid	98 ± 35	Tablets	breakfast, 162–381 kcal	↑ TSH level (by 64%)
Marina et al. [40]	14, hypothyroid	200	liquid form	breakfast, 132 kcal	no significant changes in fT4 levels
Morelli et al. [41]	59, hypothyroid	not specified	liquid form	patient’s usual breakfast	no significant changes in TSH levels
Cappelli et al. [42]	77, hypothyroid	75	liquid form	patient’s usual breakfast	no significant changes in TSH, fT4 and fT3 levels
Pirola et al. [43]	761, hypothyroid	75	liquid form	patient’s usual breakfast	no significant changes in TSH levels
Cappelli et al. [46]	1, hypothyroid	75	liquid form	Lunch	no significant changes in thyroid hormonal profiles
Cappelli et al. [47]	60, euthyroid	106 ± 24	soft gel capsules	patient’s usual breakfast	no significant changes in TSH levels, ↓ fT4 and fT3 levels (by 7% both)
Liel et al. [49]	13, hypothyroid	50–470	Not specified	fiber (whole wheat bread, bran, granola, psyllium)	↑ TSH level (ranging from 7,4 to > 50 mU/L)
Chiu et al. [50]	8, healthy	600	Tablets	fiber (psyllium)	↓ L-T4 absorption (by 8%)
Fruzza et al. [55]	1, hypothyroid	50	Not specified	soy-based infant formula	↑ TSH level (216 mU/L), ↓ fT4 level (4.0 μg/dL)
	1, hypothyroid	112	Not specified	soy-based infant formula	↑ TSH level (248 mU/L), ↓ fT4 level (<0.4ng/dL)
Conrad et al. [56]	78, hypothyroid	7.4 per kg	Not specified	soy-based infant formula	62.5% patients with TSH > 10 mU/L after 4 months
Bell et al. [57]	1, hypothyroid	200	Tablets	soy-protein containing cocktail	difficulty in suppressing TSH level
Persiani et al. [58]	12, hypothyroid	25–125	Tablets	soy-containing supplement	no significant changes in thyroid hormones levels
Chon et al. [59]	10, healthy	1000	Tablets	cow milk	↓ peak serum TT4 level by 7.8%, ↓ AUC by 8%
Benvenga et al. [61]	6, hypothyroid	200	Tablets	espresso	average T4 ↓ 36%, peak T4 ↓ 30%, tmax delayed by 38 min.
	9, healthy	200	Tablets	espresso	average T4 ↓ 29%, peak T4 ↓ 19%, tmax delayed by 43 min.
Sindoni et al. [62]	6, hypothyroid	1.6–2.2 per kg	Tablets	espresso and barley coffee	failure to normalize TSH levels
Węgrzyn [63]	1, hypothyroid	175	Tablets	drip coffee	clinical signs of hypothyroidism (TSH level—8.27 mU/L)
Vita et al. [64]	8, hypothyroid	1.6–2.8 per kg	soft gel capsules	coffee	comparable TSH levels for coffee 5 min. and 1h after L-T4
Cappelli et al. [65]	54, hypothyroid	73±14	liquid form	coffee	comparable TSH, fT3 and fT4 levels for coffee 30 min. before and with L-T4
Lilja et al. [69]	1, hypothyroid	100	Not specified	grapefruit juice	↑ TSH level (63.7 mU/L), ↓ fT4 level (6.4 pmol/L)
	10, healthy	600	Not specified	grapefruit juice	↓ AUC (by 9%), ↓ Cmax (by 11%)
Tesic et al. [71]	1, hypothyroid	200–700	Tablets	juice and mint tea	↑ TSH level (> 100 mU/L), ↓ fT4 level (5.9 pmol/L), undetectable fT3 level
Deiana et al. [72]	1, hypothyroid	1.6 per kg	Not specified	papaya	↑ TSH level (25 mU/L)
	1, hypothyroid	1.6 per kg	Not specified	papaya	↑ TSH level (from 0.8 to 15 mU/L), ↓fT3 and fT4 levels
Singh et al. [77]	20, hypothyroid	> 1 per kg	Not specified	calcium carbonate	↓fT4 level, ↑ TSH level in 20% of patients
Singh et al. [79]	7, healthy	1000	Tablets	calcium carbonate	↓ L-T4 absorption (from 83.7% to 53.7%), tmax delayed (from 2 to 4 h)
Schneyer et al. [80]	3, hypothyroid	125–325	Not specified	calcium carbonate	↑ TSH level (ranging from 7.3 to 13.3 mU/L)
Csako et al. [81]	1, hypothyroid	175–188	Not specified	calcium carbonate	↑ TSH level (41.4 mU/L)
Butner et al. [82]	1, hypothyroid	150	Not specified	calcium carbonate	↑ TSH level (21.85 mU/L)
Mazokopakis et al. [83]	1, hypothyroid	88	Not specified	calcium carbonate	↑ TSH level (9.8 mIU/L),↓fT4 level (0.2 ng/dL)
Irving et al. [11]	450, hypothyroid	not specified	Tablets	calcium carbonate	↑ TSH level (up to over 5 mU/L) in 4.4% of patients
Diskin et al. [84]	65, hypothyroid	95–98	Not specified	calcium carbonate	↑ TSH level (23.8 ± 19.5 mU/L)
Zamfirescu et al. [85]	8, healthy	1000	Tablets	calcium carbonate, calcium citrate, calcium acetate	↓ L-T4 absorption (by 20–25%)
Morini et al. [86]	50, hypothyroid	1.47 per kg	Tablets	calcium supplements	↑ TSH level (3.33 ± 1.93 mU/L)
Benvenga et al. [87]	12, hypothyroid	1.7 per kg	liquid form and tablets	calcium carbonate	↓ TSH for liquid form vs. tablet (2.15 ± 1,4 mU/L vs. 8.74 ± 7.2 mU/L)
Campbell et al. [89]	14, hypothyroid	75-150	Not specified	ferrous sulfate	↑ TSH level (from 1.6 to 5.4 mU/L)
Shakir et al. [90]	1, hypothyroid	150	Not specified	ferrous sulfate	↑ TSH level (56 mU/L), ↓ fT4 level (0,48 ng/dL)
Leger et al. [92]	1, hypothyroid	not specified	Not specified	ferrous fumarate	↑ TSH level (243 mU/L), ↓ fT4 level (<0.52 pmol/L)
Irving et al. [11]	429, hypothyroid	not specified	Tablets	iron supplements	↑ TSH level in 7.5% of patients
Benvenga et al. [87]	8, hypothyroid	1.7 per kg	liquid form and tablets	ferrous sulfate	↓ TSH for liquid form vs. tablet (1.68 ± 0.9 mU/L vs. 8.74 ± 7.2 mU/L)
Liel et al. [75]	5, hypothyroid	not specified	Not specified	aluminium hydroxide	↑ TSH level (from 2.62 to 7.19 mU/L)
John-Kalarickal et al. [98]	7, hypothyroid	1000	Not specified	chromium picolinate	↓ L-T4 bioavailability (by 17%)
Jubiz et al. [99]	31, hypothyroid	100	Tablets	vitamin C	↓ TSH level (by 69%), normalized TSH in 54.8% of patients
Antunez et al. [100]	28, hypothyroid	>1.7 per kg	Tablets	vitamin C	↓ TSH level (from 9.01 ± 5.51 mU/L to 2.27±1.61 mU/L)
Dickerson et al. [102]	13, hypothyroid	not specified	not specified	enteral nutrition	hypothyroidism subclinical (TSH—6–10 mU/L) or overt (TSH >10 mU/L)
Pirola et al. [105]	20, euthyroid	1.6 per kg	liquid form and crushed tablets	enteral nutrition	comparable thyroid hormones profile for both formulations

↑ increase ↓ decrease.

**Table 2:** Levothyroxine—food ingredients interactions - recommendations for health care professionals.

Food Interacting with L-thyroxine	Sources of Evidence	Mechanism of Interaction	Effects of Interaction	Recommendations for Health Professionals
Fiber (whole wheat bread, bran)	case series [49], non-randomized cross-over study [50]	non-specific adsorption of l-T4 to the fiber	malabsorption of l-T4, impaired efficacy of treatment	advise to separate fiber and l-T4 intake by 1 h monitor thyroid parameters more frequently, adjust L-T4 doses when needed
Soy products (soy-protein cocktails, soy-based infant formulas)	case reports [52,53,55,57], case series [54], retrospective cohort study [56]	adsorption of l-T4 on the surface of soy protein	malabsorption of l-T4, impaired efficacy of treatment	advise to separate soy protein and l-T4 intake by 1 h monitor thyroid parameters more frequently, adjust l-T4 doses when needed
Cow milk	non-randomized cross-over study [59]	probable adsorption of l-T4 on casein	impaired bioavailability of l-T4	cannot be made due to the insufficient evidence
Coffee (espresso, drip coffee, barley coffee)	case report [63], case series [61,62], uncontrolled clinical study [65], non-randomized cross-over study [64]	the sequestration of l-T4 by coffee	malabsorption of l-T4, impaired efficacy of treatment	advise to delay coffee intake by 1 h after l-T4 administration consider changing formulation from tablets to oral liquid form/gel capsules
Juice (grapefruit juice, orange juice, apple juice)	case report [69], randomized clinical trial (against interaction) [69]	blocking of OATP transporters	malabsorption of l-T4, impaired efficacy of treatment	cannot be made due to insufficient evidence advise to avoid excessive intake
Fruit (papaya)	case report [72]	unknown	malabsorption of l-T4, impaired efficacy of treatment	cannot be made due to the insufficient evidence advise to avoid excessive intake
Calcium (carbonate, acetate, citrate)	case reports [81,82,83], case series [80], retrospective cohort studies [11,84,86], prospective cohort study [77], uncontrolled clinical study [87], non-randomized cross-over study [85]	unspecific adsorption of l-T4, creating insoluble or sparingly soluble complexes in the intestine	malabsorption of l-T4, impaired efficacy of treatment	advise to delay intake by 2–4 h after l-T4 administration consider changing formulation from tablets to oral liquid form/gel capsules monitor thyroid parameters more frequently
Iron (ferrous citrate and fumarate)	case reports [89,90,91], retrospective cohort study [11,93], uncontrolled clinical study [88]	unspecific adsorption of l-T4, creating insoluble or sparingly soluble complexes in the intestine	malabsorption of l-T4, impaired efficacy of treatment	advise to delay intake by 2–4 h after l-T4 administration consider changing formulation from tablets to oral liquid form/gel capsules monitor thyroid parameters more frequently
Aluminium (hydroxide)	case reports [93,94], uncontrolled clinical study [75]	unspecific adsorption of l-T4, creating insoluble or sparingly soluble complexes in the intestine	malabsorption of l-T4, impaired efficacy of treatment	advise to delay intake by 2–4 h after l-T4 administration consider changing formulation from tablets to oral liquid form/gel capsules monitor thyroid parameters more frequently
Chromium (picolinate)	non-randomized cross-over study [98]	unspecific adsorption of l-T4, creating insoluble or sparingly soluble complexes in the intestine	malabsorption of l-T4, impaired efficacy of treatment	advise to delay intake by 3–4 h after l-T4 administration consider changing formulation from tablets to oral liquid form/gel capsules monitor thyroid parameters more frequently
Vitamin C	uncontrolled clinical study [100], non-randomized cross-over study [99]	lowering of gastric pH	enhanced absorption of l-T4	consider advising concomitant ingestion of vitamin C and l-T4

## Abbreviations

EN	Enteral nutrition
fT3	Free T3
fT4	Free T4
L-T4	Levothyroxine (L-thyroxine)
MCT	Monocarboxylate transporter family
NTCP	Sodium-taurocholate co-transporting polypeptide transporters
OATP	Organic anion-transporting polypeptide family
PDA	Prolonged Dose Adjustment
PPI	Pomp proton inhibitors
RCT	Randomized control trial
T3	Triiodothyronine
T4	Thyroxine
TFT	Thyroid function tests
TSH	Thyroid-stimulating hormone
TT4	Serum total thyroxine level
